# Saccadic Reaction Times to Audiovisual Stimuli Show Effects of Oscillatory Phase Reset

**DOI:** 10.1371/journal.pone.0044910

**Published:** 2012-10-03

**Authors:** Adele Diederich, Annette Schomburg, Hans Colonius

**Affiliations:** 1 School of Humanities and Social Sciences, Jacobs University Bremen, Bremen, Germany; 2 Department of Psychology, Carl von Ossietzky University, Oldenburg, Germany; CNRS – Université Claude Bernard Lyon 1, France

## Abstract

Initiating an eye movement towards a suddenly appearing visual target is faster when an accessory auditory stimulus occurs in close spatiotemporal vicinity. Such facilitation of saccadic reaction time (SRT) is well-documented, but the exact neural mechanisms underlying the crossmodal effect remain to be elucidated. From EEG/MEG studies it has been hypothesized that coupled oscillatory activity in primary sensory cortices regulates multisensory processing. Specifically, it is assumed that the phase of an ongoing neural oscillation is shifted due to the occurrence of a sensory stimulus so that, across trials, phase values become highly consistent (phase reset). If one can identify the phase an oscillation is reset to, it is possible to predict when temporal windows of high and low excitability will occur. However, in behavioral experiments the pre-stimulus phase will be different on successive repetitions of the experimental trial, and average performance over many trials will show no signs of the modulation. Here we circumvent this problem by repeatedly presenting an auditory accessory stimulus followed by a visual target stimulus with a temporal delay varied in steps of 2 ms. Performing a discrete time series analysis on SRT as a function of the delay, we provide statistical evidence for the existence of distinct peak spectral components in the power spectrum. These frequencies, although varying across participants, fall within the beta and gamma range (20 to 40 Hz) of neural oscillatory activity observed in neurophysiological studies of multisensory integration. Some evidence for high-theta/alpha activity was found as well. Our results are consistent with the phase reset hypothesis and demonstrate that it is amenable to testing by purely psychophysical methods. Thus, any theory of multisensory processes that connects specific brain states with patterns of saccadic responses should be able to account for traces of oscillatory activity in observable behavior.

## Introduction

Neural oscillatory activity in the brain has been shown to play a key role in cognitive performance and attentional selection in both the visual and the auditory domain [1]–[4]. An increase in synchrony within the neural representation of an object or location increases the efficacy of that neural representation at the next synaptic stage in the brain and suggests that increasing synchrony is a candidate for the neural correlate of attentional selection [5]. Specifically, high-frequency gamma rhythms (30–80 Hz) facilitate processing of stimuli in the locus of attention [6], [7], whereas oscillatory activity in the beta frequency range (13–30 Hz) has been associated with sensory-motor and integrative multisensory processing [8] (see [9] for a recent review). In addition to oscillatory power, the momentary phase of brain oscillations has been attributed a central role in attentional selection. The amplitude of faster rhythms (beta, gamma) have been found to be a function of the phase of slower oscillations, *i.e*., delta (0–4 Hz) and theta (4–8 Hz) [10]–[12]. Thus, probing of cross-frequency phase-amplitude coupling, as a mechanism to coordinate neural activity on multiple timescales and different levels of the sensory processing hierarchy, has become a major issue in many recent neurophysiological studies [12], [13].

Up to now, the primary source of evidence for the existence of neural oscillations has, quite naturally, been the recording of electrical activity through EEG measurements. Given the hypothesized implications of oscillatory activity on perceptual and attentional processes, however, it should also be possible to measure fluctuations of the associated perceptual and cognitive functions through psychophysical measures [14]. Indeed, it has been observed that phase modulation by external stimulation, e.g. a subliminal flicker cue [15] or entrainment through periodic stimulation [16], [17] speeds up and enhances target detection and discrimination, as well as saccadic reaction times (SRT) [18], at specific moments of time supporting the hypothesis that momentary phase modulates attentional selection [19], [20].

**Figure 1:**
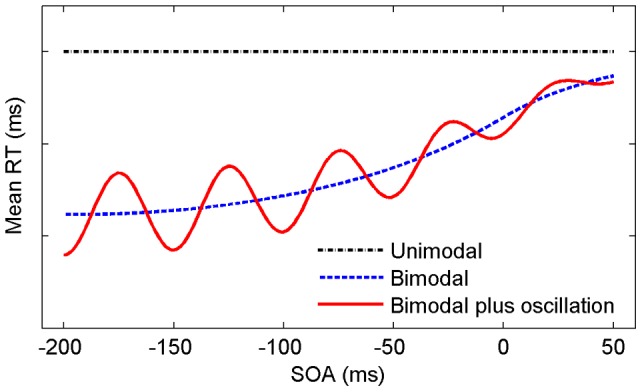
Predictions for mean bimodal RT with and without a phase reset effect as function of SOA. Horizontal (dashed) line indicates unimodal RT, bimodal (dashed/solid) line shows mean RT without/with effect of oscillatory activity (idealized functional forms).

Here we address the question of whether phase modulations evoked by crossmodal stimulation might underly multisensory integration effects in saccadic onset responses. Initiating an eye movement towards a suddenly appearing visual target is faster when an accessory auditory stimulus occurs in close spatial and temporal vicinity. This *crossmodal facilitation* of saccadic reaction time (SRT) is well-documented but many aspects of the neuronal mechanisms underlying the effect remain to be elucidated [21]–[23]. From EEG/MEG studies Senkowski and colleagues have hypothesized that coupled oscillatory activity in primary visual and auditory cortices regulates multisensory processing [24], [25]. Specifically, the phase of an ongoing neural oscillation is assumed to be shifted by the occurrence of a sensory stimulus so that phase values become highly consistent across trials (*phase reset hypothesis*). Thus, if two stimuli occur with a certain time lag, the first stimulus would reset an oscillation to its ideal phase; after reset, an input that arrives within the ideal (high-excitability) phase, even from another modality, evokes amplified responses, whereas responses to inputs arriving slightly later, during the worst phase, are suppressed [26], [27]. Lakatos and colleagues [28] suggest that, “if we identify the phase an oscillation is reset to by some external (stimulus related) or internal (motor/attention related) event and its frequency, we can predict when temporal windows of high and low excitability will occur, and thus the effect of reset oscillations on sensory inputs occurring at specific times relative to the reset. Although we cannot be certain with our methods, the onset of phase reset and evoked responses in the supragranular layers probably overlaps in the case of preferred modality stimuli, meaning that the effect of reset phase on the evoked activity would be instantaneous.”(*ibid*, p. 427). Note that the phase-reset hypothesis does not presume that entrainment by a rhythmic structure in the stimulus sequence is required for synchronization of neural oscillations.

**Figure 2:**
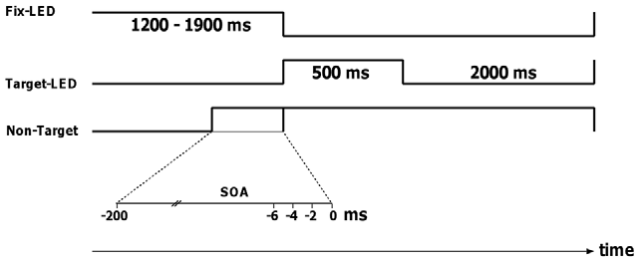
Time course of a trial. The time line indicating the SOA values (−200 to 0 in steps of 2 ms) is expanded.

Our behavioral approach is inspired by a recent measurement problem raised by Foxe and colleagues [29] (see also Discussion). They observe that phase alignment across trials may result from two different mechanisms: (1) a phase reset of ongoing oscillations or (2) a transient sensory response, which is superimposed on oscillatory activity (ibid, p. 9972). Thus, in a multisensory context when a sound and a visual stimulus are presented within the same trial, transient sensory responses are evoked in both early auditory and visual cortices making it difficult to establish, via neurophysiological measures, whether cross-sensory phase reset occurred in either cortical region.

**Figure 3:**
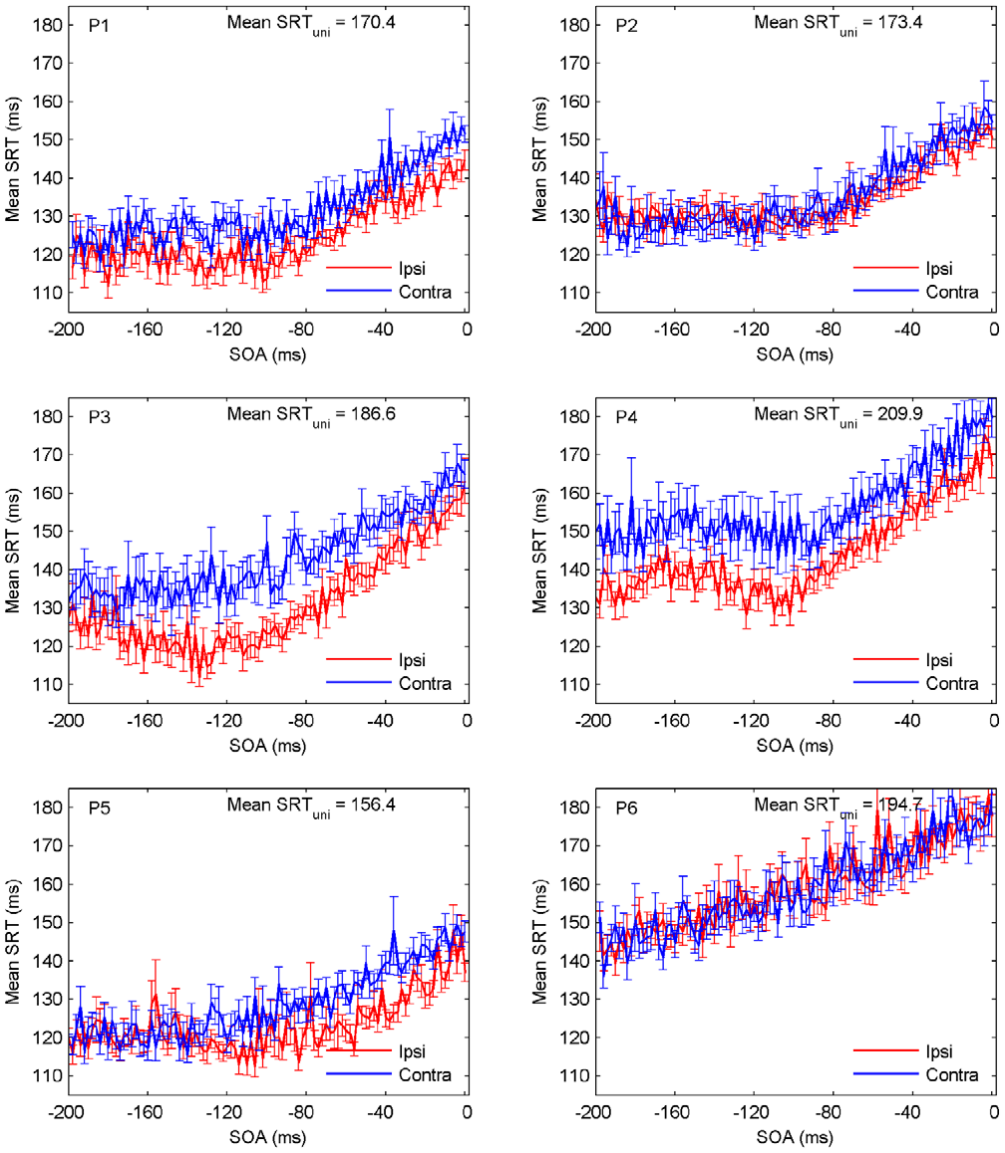
Observed mean SRT (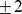 standard errors) as a function of SOA for ipsi- (red) and contralateral (blue) stimuli for all six participants. Unimodal mean SRTs are inserted as text at top.

**Table 1:** Minimum and maximum amount of multisensory response enhancement (MRE) in % for ipsi- and contralateral stimulus presentations (across all SOA values).

Participant	MRE for Bimodal Stimuli presented					
	Ipsilateral			Contralateral		
	Max	Min	Mean (median)	Max	Min	Mean (median)
1	35	15	28	31	13	24
2	28	11	24	30	9	24
3	40	13	32	31	10	25
4	39	17	33	32	13	27
5	28	5	22	26	5	19
6	29	6	19	30	10	19

VanRullen [20] has recently discussed the issue that in behavioral experiments the pre-stimulus phase will be different on successive repetitions of the experimental trial, and average performance over many trials will show no signs of modulation. Here we circumvent this problem by repeatedly presenting an auditory accessory stimulus (non-target) followed by a visual target stimulus with a specific temporal delay (stimulus onset asynchrony, SOA) between 0 and 202 ms, presented in random order in steps of 2 ms. Under the assumption that the auditory accessory reset the neural oscillation phase to about the same value in each trial, by presenting the visual target in steps of 2 ms one should map out the temporal windows of high and low crossmodal excitability. The (idealized) predictions from such a reset mechanism for mean SRTs are presented in Figure 1. The dotted (black) horizontal line indicates mean unimodal SRT to the visual target, providing a benchmark for measuring crossmodal facilitation. The dashed (blue) line shows the prediction for a visual-auditory stimulus assuming no effect of resetting, the auditory non-target being presented some SOA ms before or after the visual target. The solid (red) line illustrates the effect of high and low crossmodal excitability, due to resetting, in addition to crossmodal facilitation. Note that for most published experiments only a few SOA values are available (e.g., 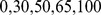 ms) and predicted curves are based on inter- or extrapolation only (e.g., [30]).

**Figure 4:**
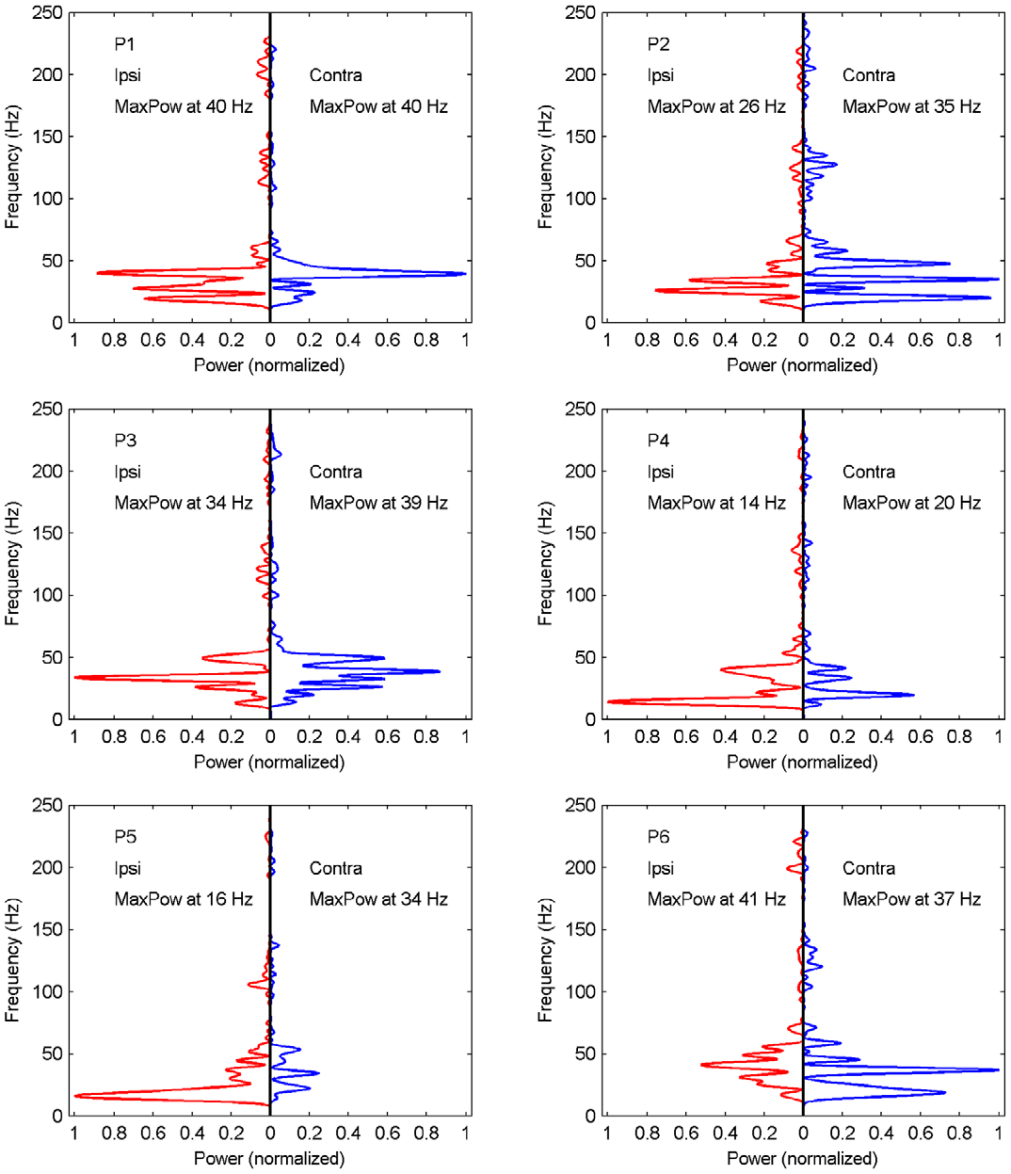
Normalized power spectra for ipsi- (left) and contralateral (right) bimodal stimulus presentation for all participants. Frequency with maximum power is indicated as text insert.

We consider average saccadic reaction time to the visual target as a sample from a discrete time series indexed by SOA. It turns out that this time series shows effects of oscillatory activity. Thus, our results support the phase reset hypothesis for crossmodal binding in a focused attention task and, moreover, demonstrate that it is amenable to testing by purely psychophysical methods.

**Figure 5:**
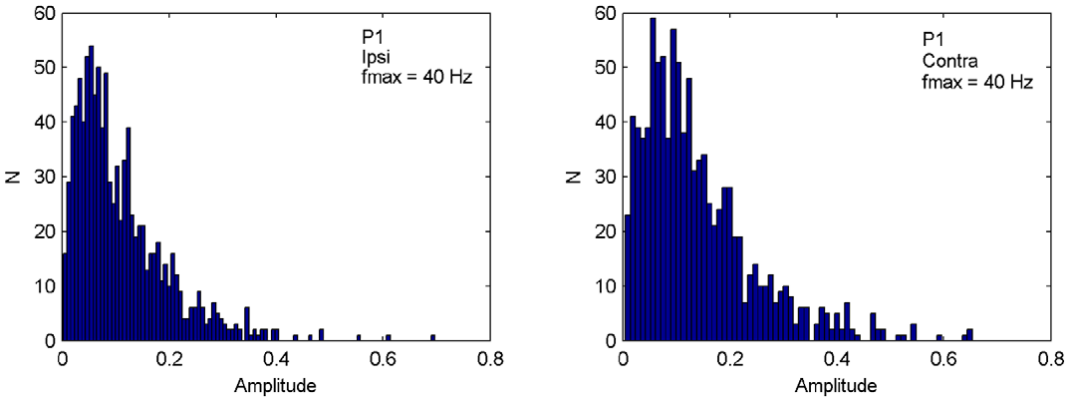
Distribution of amplitudes across shuffled time series (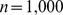) of the frequency that showed maximum amplitude (40 Hz for Participant 1) in the observed time series, for ipsi- (top) and contralateral (bottom) bimodal stimulus presentation. Observed amplitude (0.9 for ipsi-, 1.0 for contralateral) is significantly larger than those from the shuffled data series (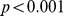). Histograms for the other participants are very similar.

## Materials and Methods

### Apparatus, stimulus presentation, and data collection

The fixation point and the visual stimuli were red light emitting diodes(LEDs) (25 mA, 5.95 mcd and 25 mA, 3.3 mcd, respectively) located on top of the speakers at the same viewing distance of 120 cm, the fixation point in the medial line and the target LEDs 20 to the left and right. Auditory stimuli were bursts of white noise (59 dB(A), rectangular envelop function) generated by two speakers (Canton Plus XS). The speakers were placed at 20 to the left and right of the fixation LED at the height of the participants' ear level and a distance of 120 cm. One PC controlled the stimulus presentation, and two other interlinked PCs controlled the EyeLink program. Control software for the stimulus presentation operated on Realtime-Linux (RTLinux), a hard real-time kernel (RTLinux patched kernel) that runs Linux as its idle thread. Signal output was carried out by a card (PCIM DDA06/16), equipped with six digital-analog converters and three digital in- and outports, which fed the control electronic with the generated time signals for the LEDs, the loud speakers and the vibration emitter, the latter not being used in the present study.

**Figure 6:**
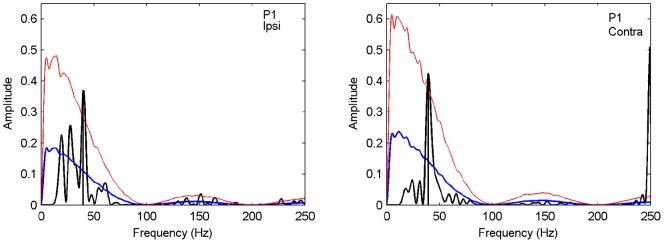
The original spectrum (black line) plotted against mean spectrum (blue line) averaged across 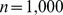 spectral samples from the set of shuffled time series. Red line indicates one-sided confidence interval bound (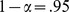). Left panel: ipsilateral; right panel: contralateral condition (Participant 1).

Participants were seated in a completely darkened, sound attenuated room with the head positioned on a chin rest, elbows and lower arms resting comfortably on a table. Although the eye movement equipment takes head movements into account, the participants were instructed to leave the head on the chin rest and not to move the head.

**Figure 7:**
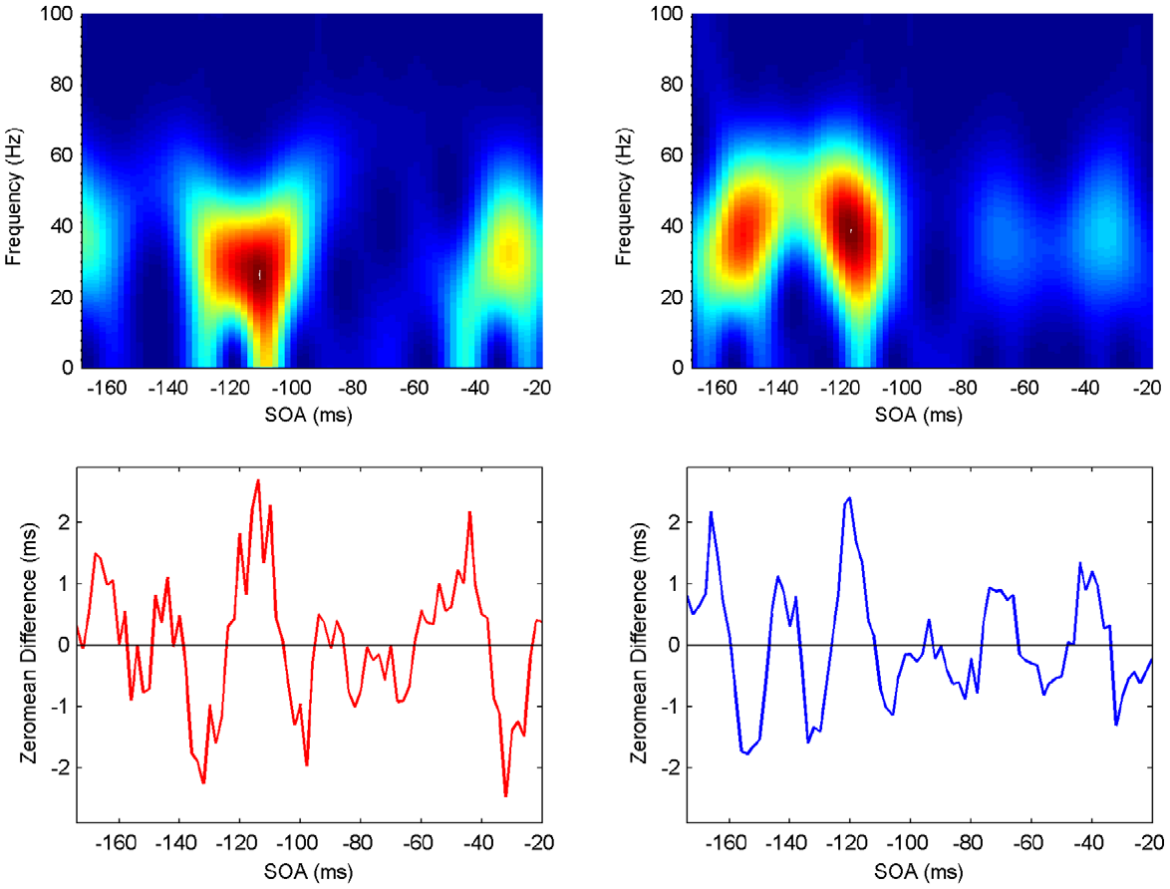
Spectrogram showing the power spectrum as a function of time for Participant 1 (upper panels: ipsilateral left, contralateral right). Maximum power (across frequencies) occurs at specific SOA (time) periods (lower panels).

Saccadic eye movements were recorded with an infrared video camera system (EyeLink II, SR Research) with a temporal resolution of 500 Hz and horizontal and vertical spatial resolution of 0.01. Criteria for saccade detection on a trial-by-trial basis were velocity (35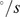) and acceleration (9,500 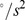). Recorded eye movements were checked for proper fixation at the beginning of the trial, eye blinks, and correct detection of start and end point of the saccade. The proportion of erroneous saccades was less than 2% in most cases (for a detailed analysis of error types, see Table S1).

**Figure 8:**
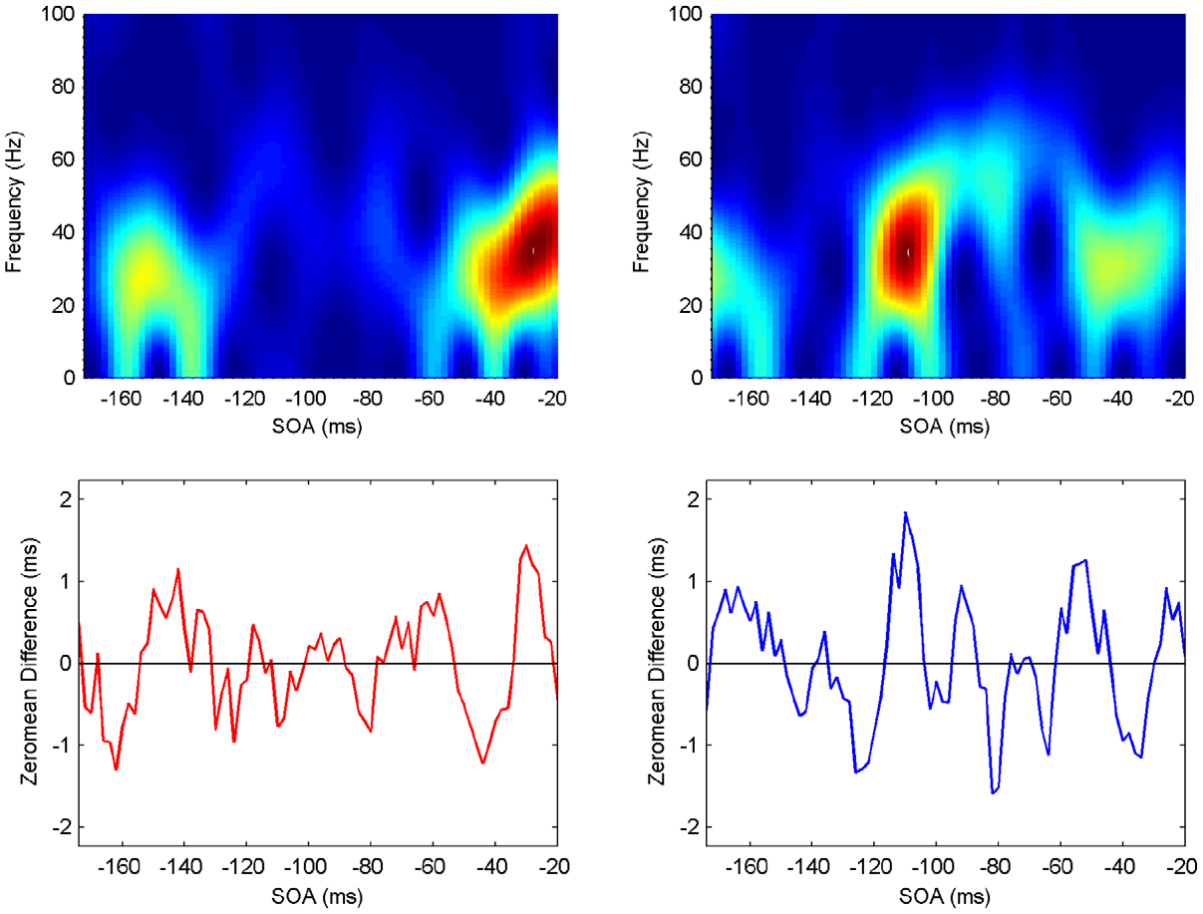
Spectrogram showing the power spectrum as a function of time for Participant 2 (upper panels: ipsilateral left, contralateral right). Maximum power (across frequencies) occurs at specific SOA (time) periods (lower panels).

### Participants

Six students, aged 20 to 27, 4 female, from Jacobs University served as paid voluntary participants. All had normal or corrected-to-normal vision and 5 were right-handed (self-description, Corens Lateral Preference Inventory, 1993). They were screened for their ability to follow the experimental instructions (proper fixation, few blinks during trial, saccades towards visual target). They gave their written informed consent prior to their inclusion in the study and the experiment has been conducted according to the principles expressed in the Declaration of Helsinki. Approval for this study was granted by the Academic Integrity Committee of Jacobs University Bremen.

**Figure 9:**
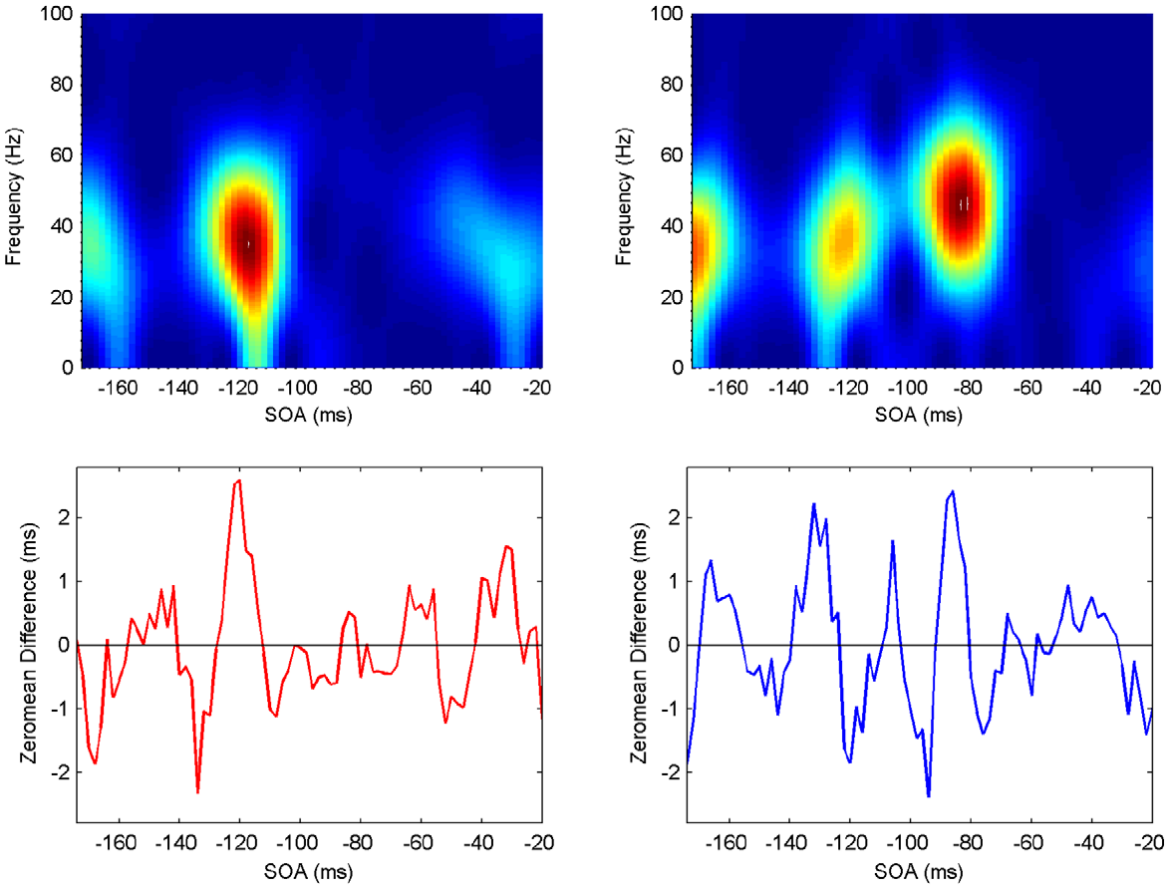
Spectrogram showing the power spectrum as a function of time for Participant 3 (upper panels: ipsilateral left, contralateral right). Maximum power (across frequencies) occurs at specific SOA (time) periods (lower panels).

### Procedure

Every experimental session began with 10 min of dark adaptation during which the measurement system was adjusted and calibrated. Each trial started with the appearance of the fixation point of random duration (1200–2100 ms). When the fixation LED disappeared, the visual target stimulus was turned on for 500 ms without a gap. Participants were instructed to gaze at the visual target as quickly and as accurately as possible and to ignore any auditory non-targets (focused attention paradigm). The visual target appeared alone or in combination with the auditory non-target in either ipsi- or contralateral position.

**Figure 10:**
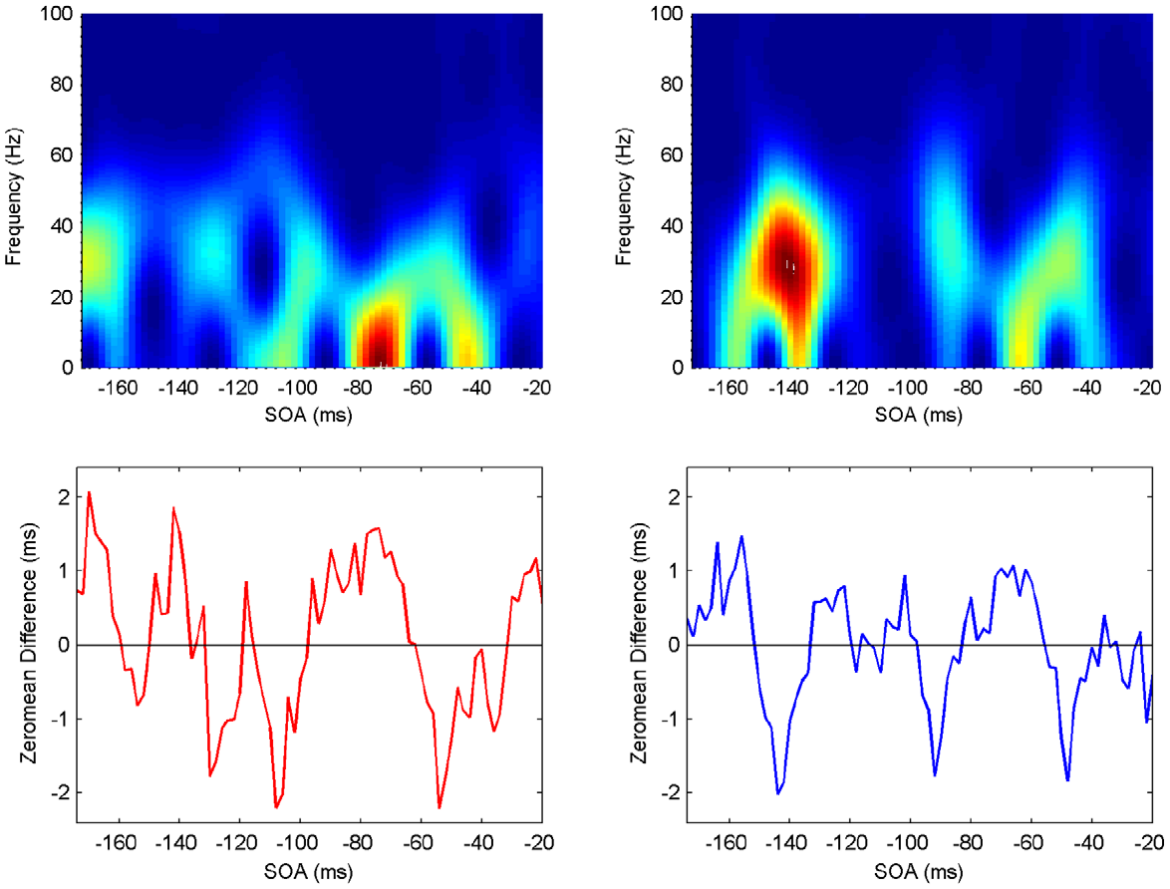
Spectrogram showing the power spectrum as a function of time for Participant 4 (upper panels: ipsilateral left, contralateral right). Maximum power (across frequencies) occurs at specific SOA (time) periods (lower panels).

The onset of the auditory non-targets was varied, in random order, between 202 ms and 0 ms prior to the target in steps of 2 ms, resulting in a total of 102 SOAs (Figure 2). The non-targets were turned off simultaneous with the visual stimulus. Thus their duration varied between 702 and 500 ms. Stimulus presentation was followed by a break of 2 s in complete darkness, before the next trial began, indicated by the onset of the fixation LED.

**Figure 11:**
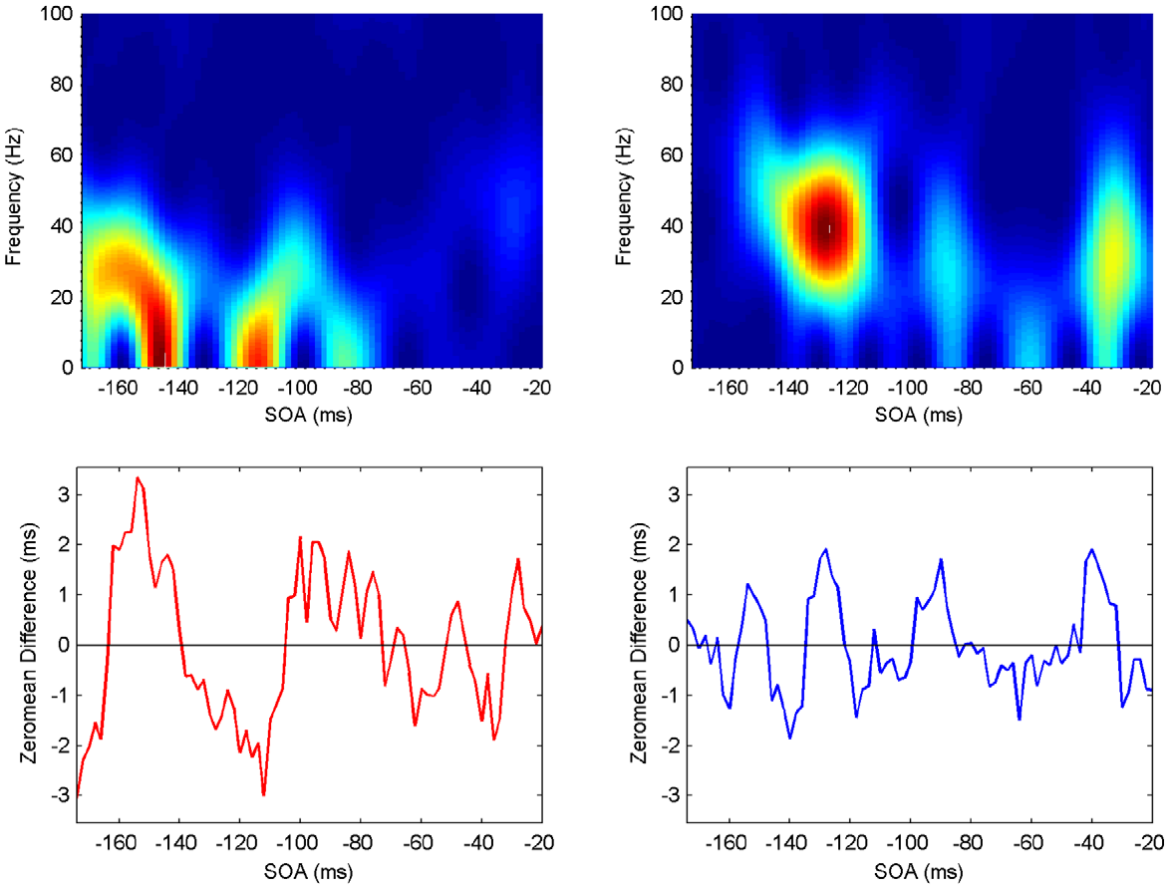
Spectrogram showing the power spectrum as a function of time for Participant 5 (upper panels: ipsilateral left, contralateral right). Maximum power (across frequencies) occurs at specific SOA (time) periods (lower panels).

One experimental block consisted of 212 trials (204 bimodal, each SOA presented once ipsi- and contralateral, 8 unimodal) randomized over SOA and laterality. Each participant performed 48 blocks, four blocks of trials within one experimental session lasting for about one hour. Each participant was engaged for about thirteen hours (twelve experimental and one training hour) and completed a total of 10,176 experimental trials.

**Figure 12:**
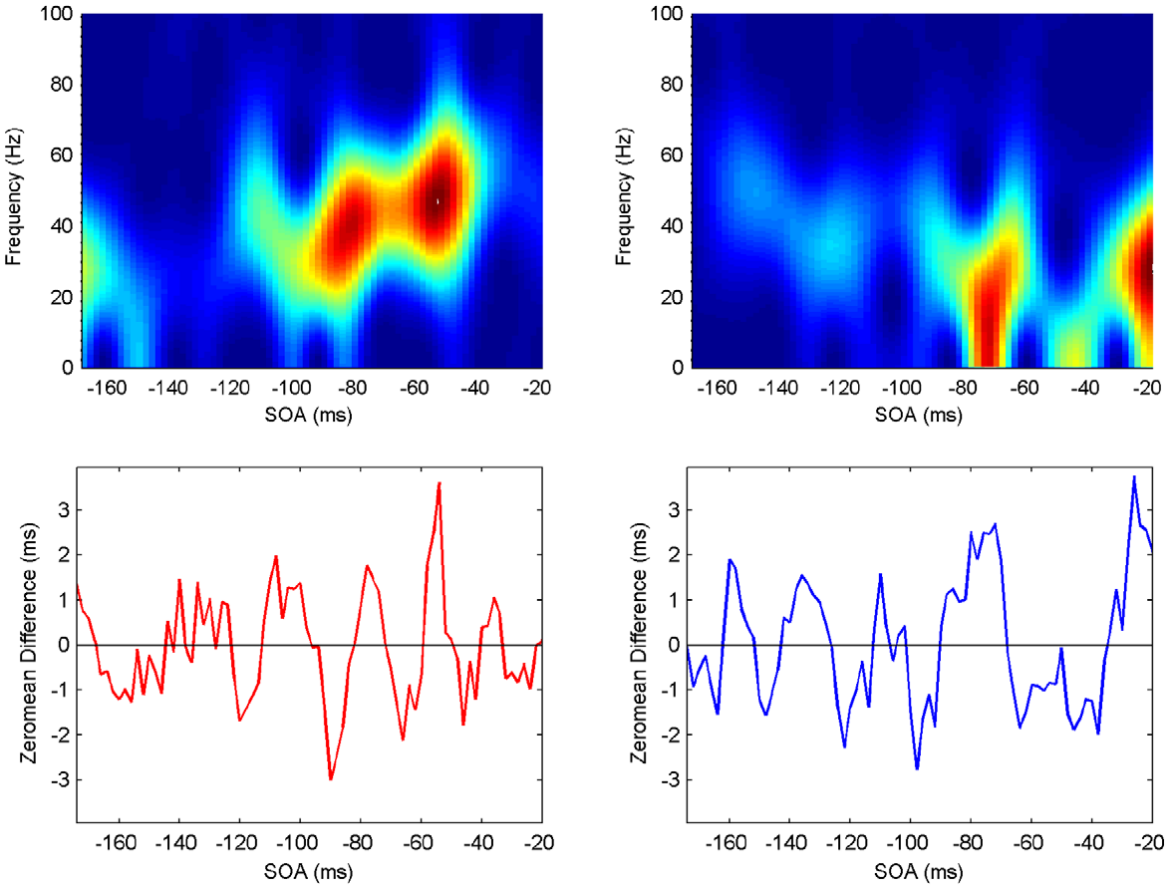
Spectrogram showing the power spectrum as a function of time for Participant 6 (upper panels: ipsilateral left, contralateral right). Maximum power (across frequencies) occurs at specific SOA (time) periods (lower panels).

### Data Analysis

For each subject, mean saccadic reaction time was analyzed as a discrete time series, considered as a function of the SOA values (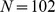), separately for the ipsi- and contralateral presentations. Prior to subjecting the data to a spectral analysis, all time series underwent some preprocessing, as described next.

### Preprocessing

#### Trend removal

It is well known that mean bimodal SRT in a focused attention paradigm exhibits an overall trend with varying SOA: it typically first decreases and then increases with the (leading) nontarget being presented closer and closer relative to the target (see e.g. [31]). Because such a trend can completely nullify the estimation of low frequency spectral content (cf. [32]), it was removed as follows. Each time series was assumed to be decomposable into two components.
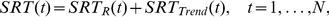
(1)where 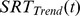 is the trend component to be eliminated and 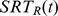, with zero mean, contains the remaining constituents of the observed mean SRT including oscillation to be subjected to further data analysis. The trend function was estimated by least-squares fitting of a 6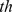-degree polynomial function to 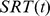 (using MATLAB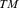 functions *polyfit* and *polyval*). Note that the polynome was chosen based on face validity by a stepwise increase of its degree.

#### Simple moving average

The stimuli were presented in SOA steps of 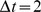 ms, which is equivalent to a sampling rate of 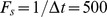 Hz. The largest frequency detectable in the data is determined by the sampling rate 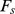, i.e., 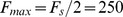 Hz. (Nyquist sampling theorem). Because, given the results reported in the EEG studies and single cell recordings mentioned in the introduction, we do not expect frequencies above 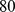 Hz, we applied a simple moving average filter to the time series 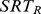 in order to remove faster fluctuations. Specifically, each point in the filtered time series, 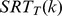, was calculated as
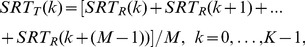
(2)where 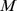 is the filter length. With 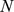 data points in the original data series the filtered data series has 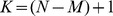 data points. A cut-off frequency of around 80 Hz requires a window length of 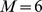 (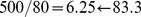 Hz cut-off frequency), resulting in 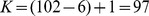 data points in the filtered series (SOA: 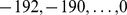)) encompassing 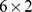
ms 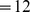 ms.

The smallest frequency that can be detected in the data, i.e., the frequency resolution, is determined by the record length 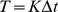. Since the filtered data series has a record length of 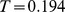 s, the frequency resolution is 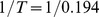 s 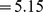 Hz. That is, only frequencies within the range of about 5 Hz to about 80 Hz are considered here. This excludes possible low frequencies bands, in particular delta and lower theta waves. However, since oscillatory activities in these bands have been associated with higher cognitive processes, such as motivation, reward, memory and emotion (see [24] for a summary), we consider the exclusion of frequencies smaller than 5 Hz not critical for the present study.

### Spectral Analysis: Discrete Fourier Transform and Spectrogram

The preprocessed discrete time series data, for each subject and for both ipsi- and contralateral presentations, were probed for their spectral components. The power spectrum is a convenient way to show how much of a signal is at a specific frequency.

On the filtered, zero-mean data series 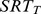 we performed a spectral analysis to separate data series into different periodic components. Note that this technique is purely descriptive to discover cyclical phenomena. The Discrete Fourier Transform (DFT) decomposes 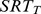, the input signal in the time domain, into an output signal in the frequency domain 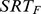, containing estimates of the amplitude and phase of the sinusoidal components, i.e.,
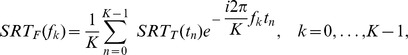


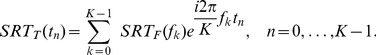
(3)


Both domains contain exactly the same information, only in different forms. The DFT was carried out by MATLAB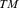 function *dft* using a zero padding methods. That is, the time series was padded with zeros to increase the number of sampling points from 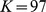 to 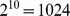 sampling points. Thereby, the frequency resolution was enhanced from 5.15 Hz to 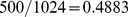 Hz. The absolute value (magnitude) of the Fourier coefficients represents the amplitude of the spectral components. Typically, the complex spectrum is presented as a one-sided power spectrum. (The complex spectrum of a real-valued signal is symmetric, containing spectral components for both, positive and negative frequencies. The components of the negative frequencies are added to the corresponding positive parts.)

In order to investigate how the frequency components identified in the DFT vary over time, i.e., as a function of SOA, we performed a *spectrogram analysis*. A spectrogram is a time-frequency presentation of a signal and is created by using a short-time Fourier transform. The analysis was carried out on the preprocessed time series utilizing the MATLAB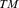 function *spectrogram*. It requires dividing the data into segments allowing an overlap of data from each segment. For each segment a Fourier transform is calculated so that the spectra are presented as a function of time. Here we set the number of segments to 20 with an overlap of 19 samples. Note that this results in an SOA range of 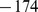 to 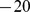 ms. Increasing the number of segments leads to a shrinkage of the time range, whereas decreasing it leads to less distinct frequencies. The choice of 20 seemed to be a good compromise. As before, the number of sampling points to calculate the discrete Fourier transform was set to 1024 points; the sampling rate was 500 Hz.

## Results

### Crossmodal Facilitation of Saccadic Reaction Time

Participants differ with respect to unimodal visual saccadic response speed by up to 50 ms. Mean saccadic reaction times to bimodal stimuli are up to 60 ms shorter than to the unimodal stimuli for all participants. Specifically, responses tend to speed up with the (leading) auditory nontarget being presented earlier relative to the visual target, and 4 out of 6 participants exhibit a typically observed spatial effect, i.e., faster responses to the ipsilateral configuration (e.g., [33]). Figure 3 shows mean saccadic response times, including error bars, as a function of SOA for all participants. Note that all graphs show a considerable fluctuation of mean SRT from one value of SOA to the next. Whether or not these fluctuations contain traces of oscillatory activity is the question being addressed in the next section.

To quantify the observed amount of facilitation we calculated a measure of multisensory response enhancement (*MRE*) relating mean SRT in the bimodal conditions to that in the unimodal condition [34],
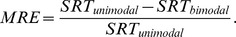



A summary, showing the minimum, maximum, and median relative amount of facilitation across all SOAs for each participant separately, is provided in Table 1.

The pattern of facilitation varies across participants. Data from subjects P2 and P6 do not show a spatial effect (ipsilateral faster than contralateral), as already suggested in Figure 3.

### Spectral Analyses

#### Power Spectra

After preprocessing, i.e., trend removal and low-pass filtering, the resulting time series underwent spectral analyses, separately for each participant and ipsi- and contralateral stimulus presentation. Figure 4 shows the power spectra normalized across ipsi- and contralateral condition. Distinct peak spectral components can be observed for both spatial conditions across all participants. For all participants maximum power is observed primarily between 20 and 40 Hz, equivalent to an oscillation with period lengths of 25 to 50 ms. Frequencies with maximum power for ipsi- and contralateral presentations (indicated in the graphs) are correlated (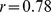, across participants). For 4 out of 6 participants maximum power was larger for ipsilateral stimuli whereas P2 and P6 show larger peaks for contralateral stimuli, a pattern that parallels the finding for those subjects of no discernible spatial effect.

Given that observed mean SRTs differ across participants, the occurrence of peak spectral components in a relatively narrow frequency range suggests the existence of an underlying oscillatory activity in the high-beta/gamma range. In order to hedge against the possibility of artifacts due to the antecedent numerical procedures we performed the same analyses as on the original data but under random permutations of the time points. If the spectral analysis results of the original data are not significantly different from those under random permutations of the time points, then our hypothesis of an underlying oscillatory activity in modulating crossmodal interaction would not be supported by the observed data.

Specifically, we first considered how the amplitude of the frequency component that was maximal in the original time series was distributed across the power spectra generated from 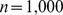 shuffled time series that were randomly drawn from the set of all 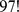 permutations. However, because frequency resolution is limited to about 5 Hz, the spectra from the DFTs on the shuffled data may not contain power at the exact maximum frequency. Therefore, the amplitude at the maximum frequency was merged with the amplitudes occurring for 10 evenly spaced frequency levels around it within a 5 Hz range. Depicting the resulting distribution of amplitudes Figure 5 shows that the amplitude of the peak frequency in the observed time series is significantly larger than those from the shuffled time series (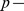
values smaller than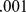).

As an additional test, we compared the spectrum of the original time series with the average spectrum across all 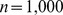 shuffled time series. Figure 6 depicts the average spectrum of the shuffled time series with the corresponding (one-sided, 95%) confidence bound calculated from the original spectrum for Participant 1. The original spectrum has its maximum peak not falling within the confidence bound, thus corroborating the previous result. For the other participants, 6 out of 10 conditions also have significant results (see Figures S1 and S2).

#### Spectrograms

The last step in the analysis is to compute spectrograms showing how the power spectrum changes over time, that is, in an SOA range of 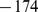 to 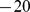 ms. Although there is a moderate level of variability, all participants exhibit clear periods of elevated power in the beta/gamma frequency range, with similar patterns but at different SOAs (Figures 7, 8, 9, 10, 11, 12). (Note: Computing spectrograms on the data before trend removal was performed yielded very similar results.) Note that, under the phase reset hypothesis, such periods of high-excitability, embedded in periods of low-excitability, are to be expected.

Given our limited range of SOA values, probing for frequency components below 5 Hz was not feasible. However, in a separate analysis, presented in the supplement, we found spectral peaks in the high-theta range (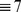 Hz) and the alpha range (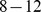 Hz) with corresponding spectrograms showing some patterns of periodicity as well (see Text S1 and Figures S3, S4, S5, S6).

## Discussion

We presented a supra-threshold auditory accessory stimulus (non-target) followed by a visual target stimulus at a specific stimulus onset asynchrony (SOA) that varied randomly between 0 and 200 ms in steps of 2 ms. Mean saccadic reaction time to the crossmodal stimulus exhibited a speedup of responses (facilitation) of up to 50 ms compared to responses to the unimodal visual stimuli. This result replicates findings from numerous studies where, however, crossmodal stimuli are typically presented using only a few SOA values with temporal spacings of 50 or 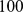 ms (e.g., [35], [31],[34]). In [33] we have shown that a multisensory integration mechanism can be distinguished empirically from an expectation/warning mechanism for this effect when one considers SOA values larger than those employed in the present study. Moreover, the difference between response speed to ipsi- vs. contralateral presentations can be described within an optimal time-window-of-integration model that features prior probabilities for the spatial coincidence of auditory and visual events ([36]).

The phase-reset hypothesis for multisensory integration holds that crossmodal interaction is evoked by the occurrence of a sensory stimulus shifting the phase of an ongoing neural oscillation to a specific value such that the processing of a subsequent stimulus in another modality is either suppressed or facilitated, depending on the exact relation between the phase of the neural oscillatory activity and occurrence of the second stimulus. The sequence of mean crossmodal saccadic reaction times was analyzed as a sample of a discrete time series indexed by the SOA values. After preprocessing (trend removal and smoothing by simple moving average) the time series was subjected to a discrete Fourier analysis. For all 6 subjects the resulting power spectra showed distinct peak spectral components within a frequency range of 20 to 40 Hz. Subsequent statistical tests, comparing the observed results with those obtained from random shuffling of the time points, supported the significance of the observed peaks. Spectrograms showed that periods of elevated oscillatory activity often seem to occur at time periods consistent with the observed frequencies of the peaks in the power spectra. Specifically, they tend to be present at SOA values that are multiples of those predicted by the peak frequencies but this needs proper statistical back-up – ideally by developing a quantitative model that would predict the periodicity.

Overall, our results provide statistical evidence for the existence of distinct peak spectral components in the time series of saccadic RTs defined across the SOAs between the onset of a nontarget auditory stimulus and a subsequent visual target. Moreover, the frequency of these periods falls within the beta and gamma frequency range of neural oscillatory activity previously observed in neurophysiological studies of multisensory integration [8]. Thus, our findings support the hypothesis that a phase-reset mechanism may underly –at least in part– the generation of multisensory interaction in saccadic speed. Although behavioral data cannot provide direct evidence that the auditory accessory resets the neural oscillation phase to about the same value in each trial, it would be difficult to account for the observed spectral distributions without this assumption. Still, there has been some discussion on whether the observed phase locking to the auditory stimulus is indeed the result of a hypothesized phase reset of ongoing oscillations, or whether this activity is being evoked by the auditory stimulus itself ([29], [37]). As noted in [38], these two alternative explanations may not be “unequivocally dissociable on empirical grounds” (ibid, p. 811).

The recent study by Romei et al. [38] provides further support for the phase reset hypothesis. Similar to the temporal setup of our study, these authors presented brief sounds followed by occipital transcranial magnetic stimulation (TMS) with an SOA range from 30 to 300 ms in steps of 15 ms. Phosphene perception rate against time postsound showed a periodic pattern at about 10 Hz phase-aligned to the sound, and this periodicity also showed up in concurrently recorded EEG measures (note that this alpha frequency is consistent with our findings presented in the supplement). These authors address the question of whether their results can be explained by the sound merely serving as a warning signal to produce temporal expectancies about the occurrence of the subsequent stimuli (Note that this issue has also been raised by one of the reviewers of this paper). Although both here and in the Romei et al. study, the different time intervals after sound onset were equally likely, in principle it cannot be ruled out that participants might estimate a central tendency of the distribution of SOAs. However, in neither study it is obvious how this would explain the occurrence of the observed periodicity.

The reset hypothesis was also corroborated recently by another psychophysical study of Fiebelkorn et al. [29] using a crossmodal stimulus arrangement similar to ours. In a visual detection paradigm, a sound was presented at the beginning of each trial, and a near-threshold visual stimulus was presented either with the sound or at 1 to 12 different times points at 500 ms intervals up to 6 s after the sound. A spectral analysis of the hit rates across the different time points revealed that “...performance on a visual-target detection task waxed and waned in a periodic fashion, time-locked to a temporally informative sound.” (ibid, p. 9978). Given the large difference in the time range between the two studies, however, no meaningful comparison between the observed oscillation frequencies can be made.

There have been many earlier attempts to find evidence for oscillatory phenomena in reaction time distributions, e.g., [39]–[42]. Unfortunately, searching for periodicities in the reaction time frequency histogram is prone to statistical artifacts [43] and, most important, does not account for the problem that the pre-stimulus phase will be different on successive repetitions of the experimental trial [20]. Nevertheless, evidence in favor of the effect of phase on saccadic speed was found in a recent study by Drewes and VanRullen [18]. Partitioning saccadic reaction time distribution into quintiles, they observed a highly significant relation between SRTs and pre-stimulus oscillatory phase of the EEG activity. Although this study did not investigate multisensory integration effects, the authors suggested a scheme that would be in sync with the phase-reset hypothesis: the precise pre-stimulus phase does not determine a single specific instant of time at which a response could be initiated but “rather a number of successive `temporal windows of opportunity” for reaction time generation, recurring at a specific frequency” (*ibid*, p. 4706).

Further support for a phase reset mechanism was found in a recent combined RT-EEG study [30]. In an auditory frequency discrimination task, Thorne and colleagues measured significant increases in the phase concentration of alpha and theta frequency activity when a visual stimulus preceded auditory stimuli between 30 and 75 ms. In accordance with Schroeder et al. [25], [26], they conjectured that a phase reset occur in auditory cortex triggered by the visual input arriving in A1 slightly before auditory inputs. Note that in contrast to the experimental paradigm used here, (i) they presented visual stimuli prior to auditory and (ii) subjects had to focus on the auditory modality rather than the visual. Resulting average manual reaction times were more than twice as long as those observed in the eye movement paradigm used here. This strongly suggest that the underlying phase reset mechanisms may not be the same, indicated also by the lower oscillatory frequencies found in their study.

Additional evidence was gathered in another combined RT-EEG study by Naue and colleagues [44]. Somewhat complementary to [30], auditory stimuli were presented from 40 to 70 ms (in steps of 5 ms) prior to a visual stimulus discrimination task. The amplitude of the beta response (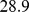 Hz) was found to be modulated by SOA. Drawing upon the phase reset hypothesis, this was considered to exhibit an effect of an auditory evoked oscillatory response in visual cortex alternating between states of high and low excitability. In correspondence to our study, manual reaction times decreased with increasing SOAs, but no oscillatory effect could be discerned in their experiment. This may be due to the SOA range being much shorter (40–70 ms vs. 0–200 ms) and the steps being larger (5 vs. 2 ms).

Although the finding of peak frequencies in the high beta/gamma range in our discrete time series is roughly consistent with those observed in neurophysiological recordings, it is clearly not a straightforward task to determine the exact correspondence between neural oscillatory activity and periodicities in perceptual performance. As observed in a recent review by Siegel and colleagues [9], “...different frequency bands have been implicated in the same cognitive process and different cognitive processes seem to involve identical frequency ranges” (ibid, p. 127). For the area of multisensory integration, at least, the results of this study imply that any theory of multisensory processes that connects specific brain states with patterns of saccadic responses must be able to account for traces of oscillatory activity in the observable behavior.

## Supporting Information

Figure S1
**The original spectrum plotted against confidence interval bounds (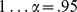) for amplitude distribution across frequencies (Participants 2–4).** Means are computed across n = 1; 000 samples from the set of shuffled time series, standard errors are calculated from original time series (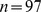).(TIF)

Figure S2
**The original spectrum plotted against confidence interval bounds (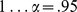) for amplitude distribution across frequencies (Participants 5–6).** Means are computed across 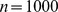 samples from the set of shuffled time series, standard errors are calculated from original time series (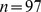).(TIF)

Figure S3 **Procedure for determining lower frequencies.** The observed mean SRT with its trend function, a polynomial of degree 6 (left upper panel); the trend (black) of the trend function (red), a polynomial of degree 2 (upper right); the different between both trend functions, zero-mean difference function (lower left); power spectrum of the zero-mean difference function (lower right).
(TIF)

Figure S4 **Power spectra for Participants 1–6 (lower frequencies).**
(TIF)

Figure S5 **Spectrograms for participants P1, P2, and P3.** Left panels: Ipsilateral presentation. Right panels: contralateral presentation.
(TIF)

Figure S6 **Spectrograms for participants P4, P5, and P6.** Left panels: Ipsilateral presentation. Right panels: contralateral presentation.
(TIF)

Table S1 **Percentage of errors by type for each participant.**
(PDF)

Text S1 **Probing for slower oscillatory activity.**
(PDF)
