# Supplementary figures and images for: Saccadic Reaction Times to Audiovisual Stimuli Show Effects of Oscillatory Phase Reset
Source: PLoS One. 2012 Oct 3;7(10):e44910. doi: 10.1371/journal.pone.0044910 (PMC3463580; doi:10.1371/journal.pone.0044910)

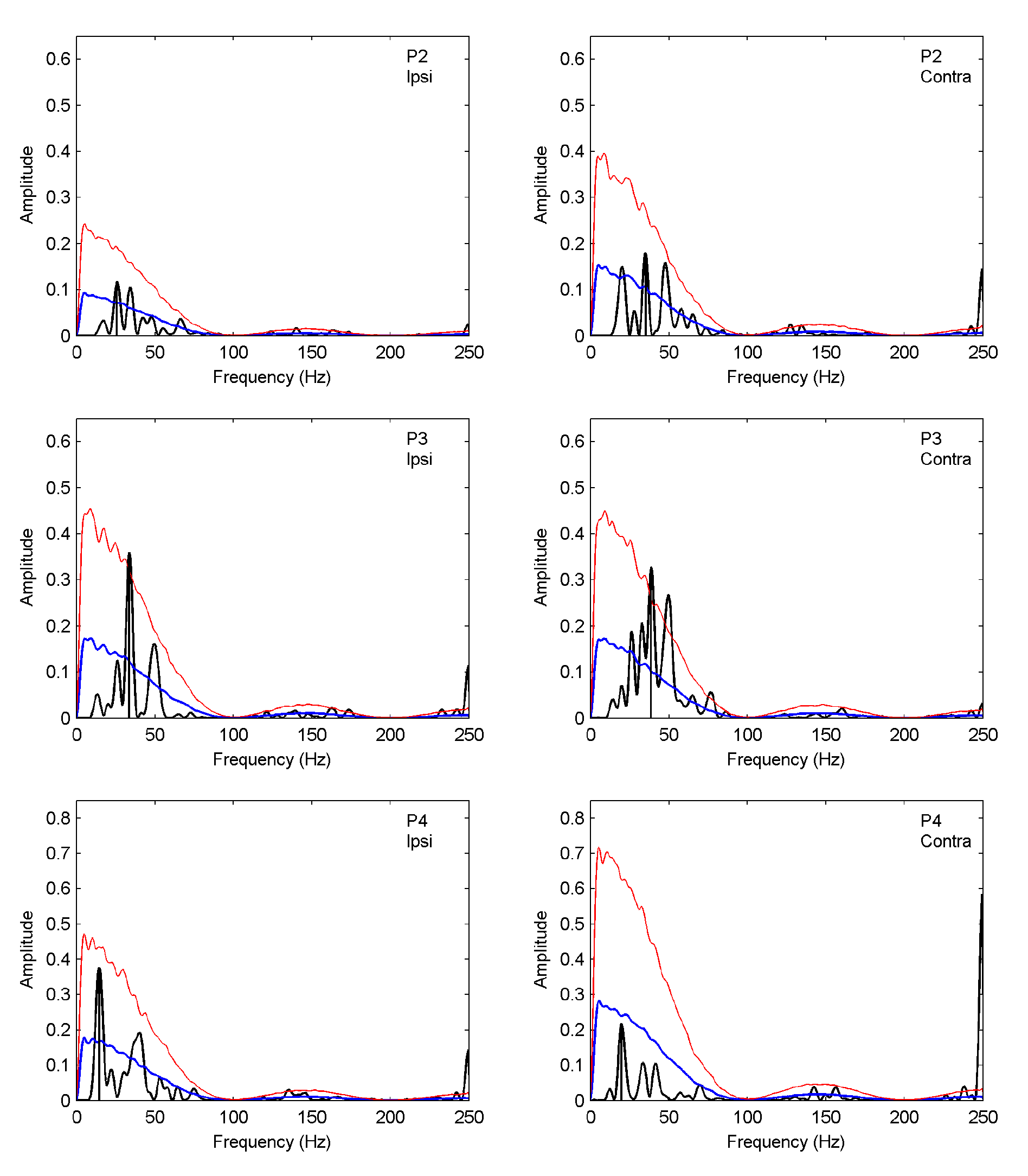

Supplement: Figure S1 — The original spectrum plotted against confidence interval bounds () for amplitude distribution across frequencies (Participants 2–4). Means are computed across n = 1; 000 samples from the set of shuffled time series, standard errors are calculated from original time series (). (TIF) [file pone.0044910.s001.tif]

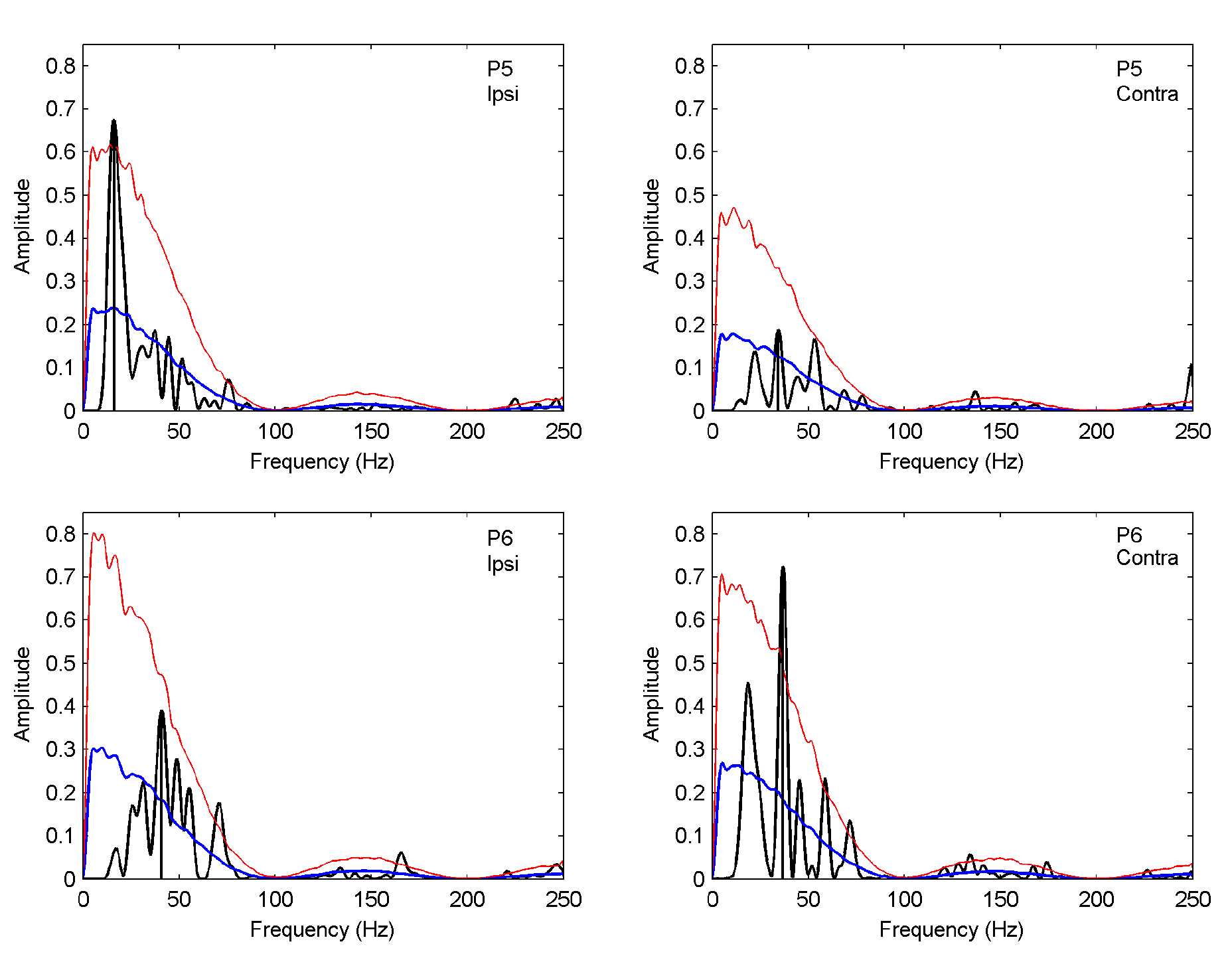

Supplement: Figure S2 — The original spectrum plotted against confidence interval bounds () for amplitude distribution across frequencies (Participants 5–6). Means are computed across samples from the set of shuffled time series, standard errors are calculated from original time series (). (TIF) [file pone.0044910.s002.tif]

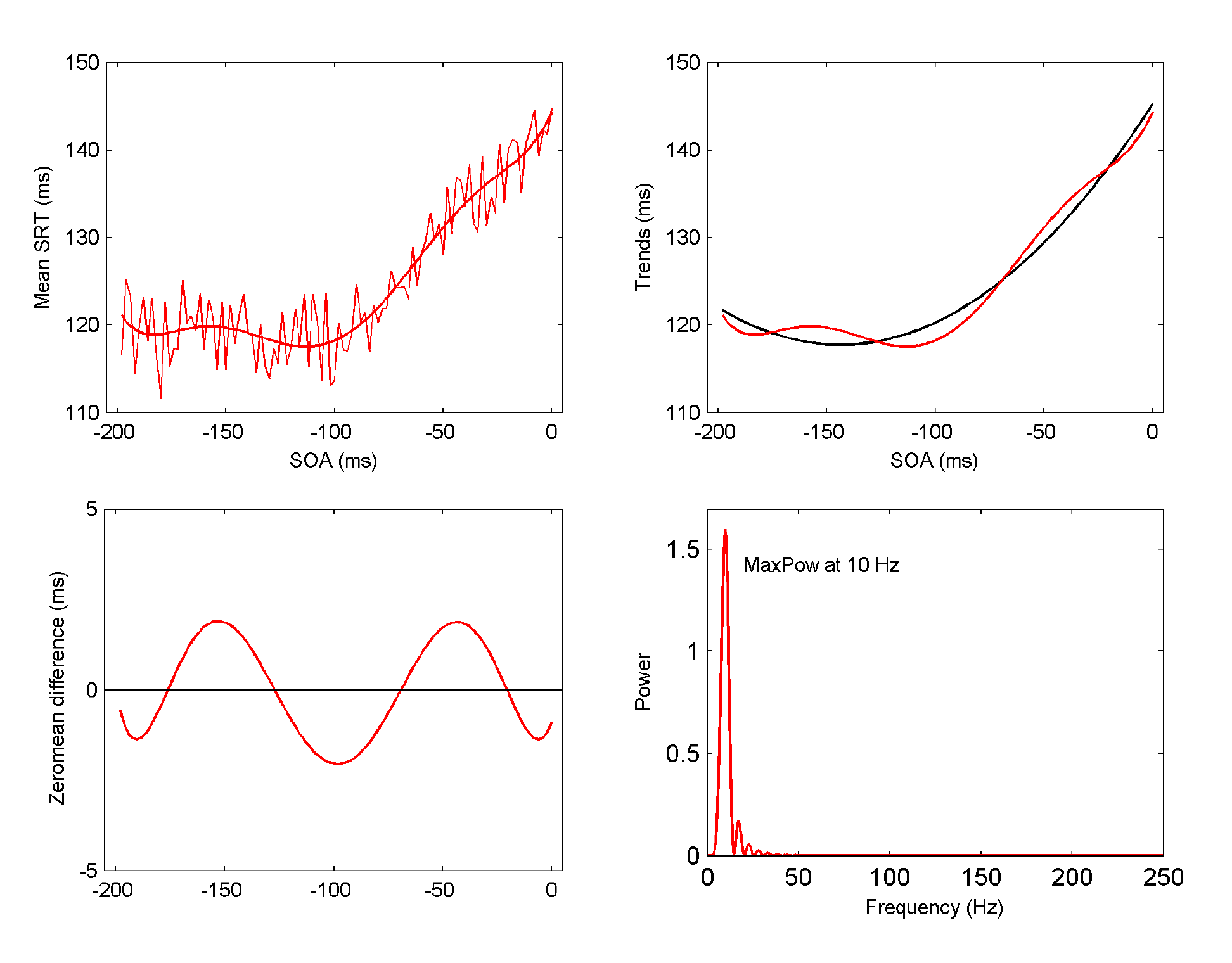

Supplement: Figure S3 — Procedure for determining lower frequencies. The observed mean SRT with its trend function, a polynomial of degree 6 (left upper panel); the trend (black) of the trend function (red), a polynomial of degree 2 (upper right); the different between both trend functions, zero-mean difference function (lower left); power spectrum of the zero-mean difference function (lower right). (TIF) [file pone.0044910.s003.tif]

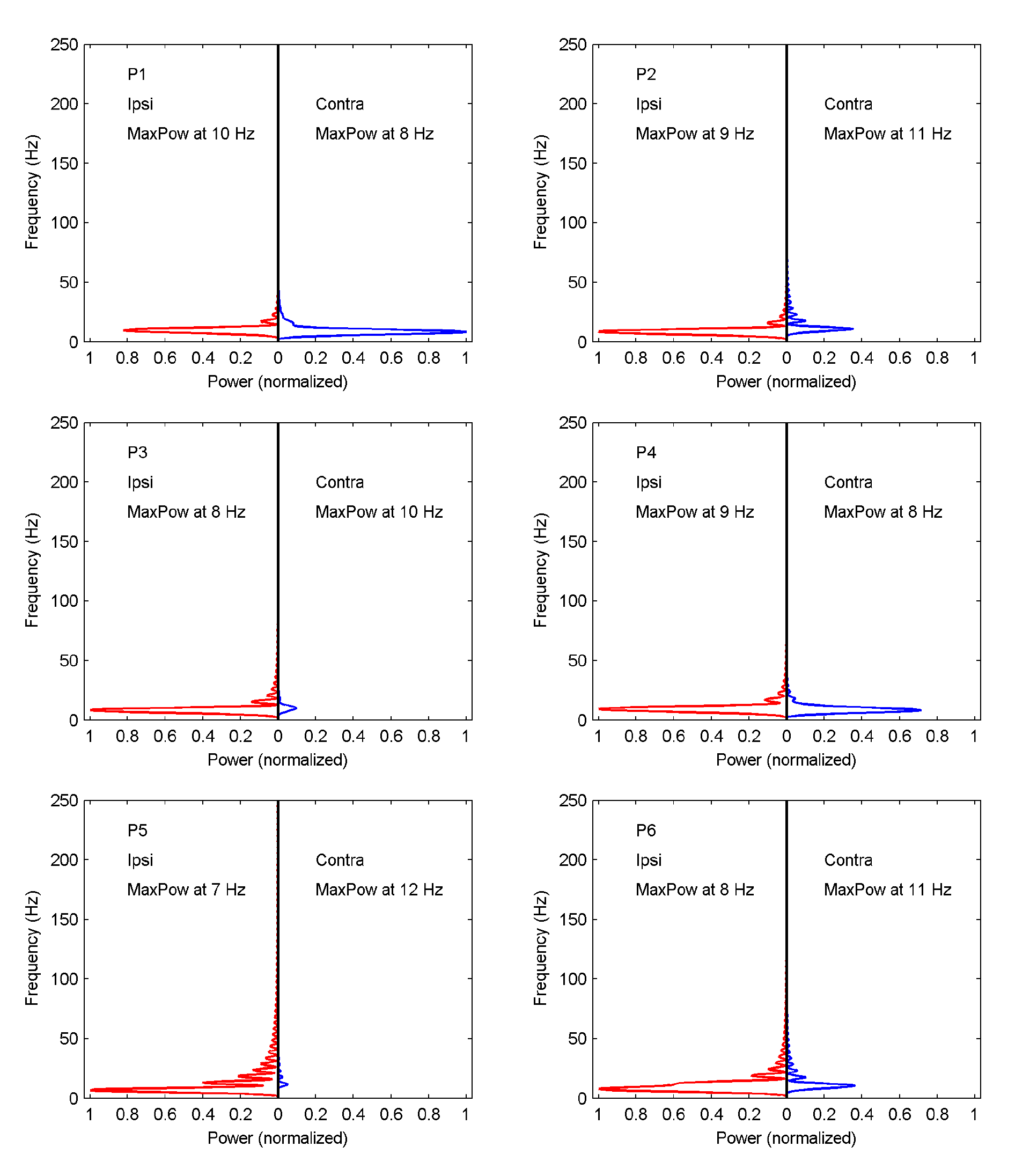

Supplement: Figure S4 — Power spectra for Participants 1–6 (lower frequencies). (TIF) [file pone.0044910.s004.tif]

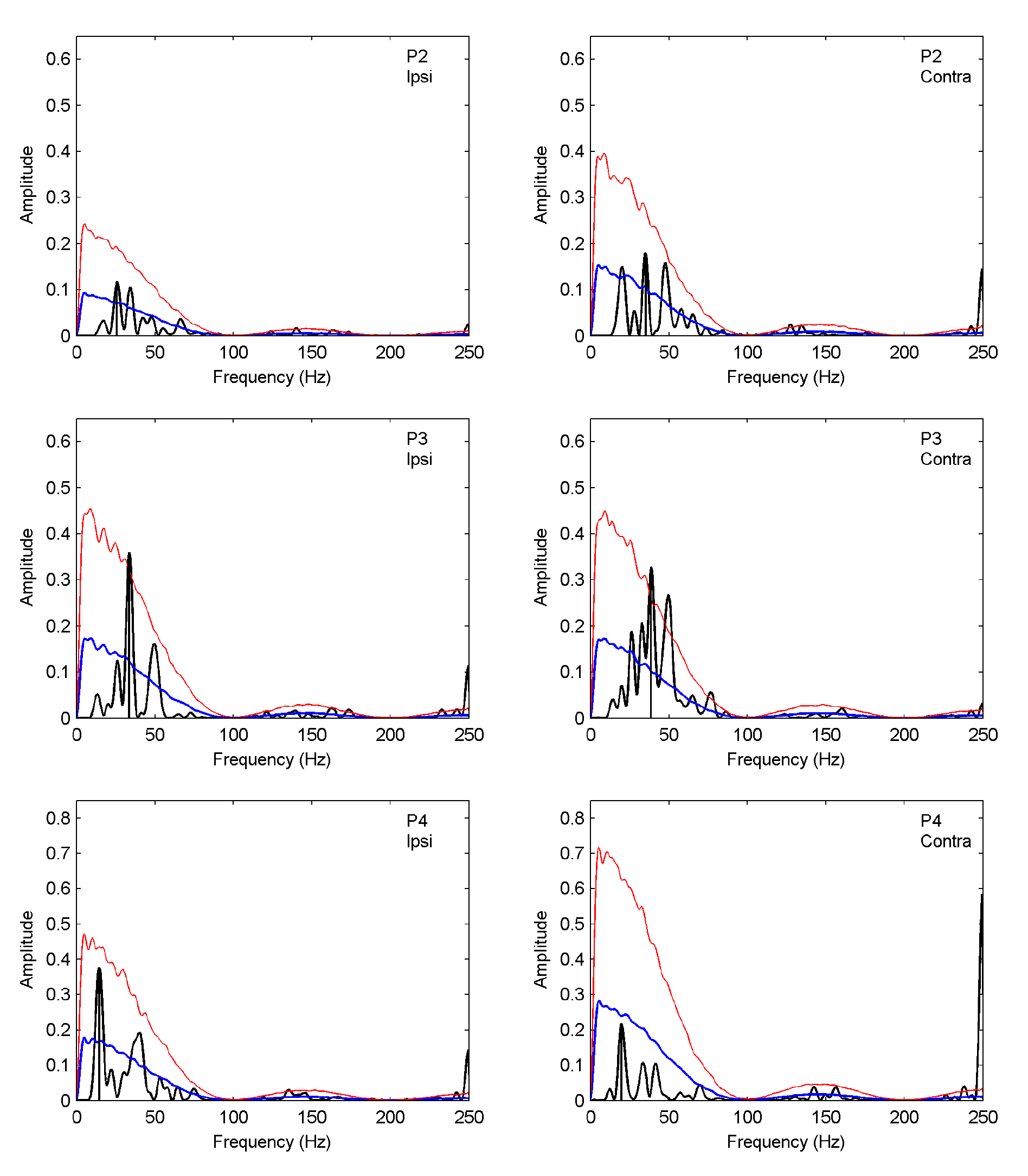

Supplement: Figure S5 — Spectrograms for participants P1, P2, and P3. Left panels: Ipsilateral presentation. Right panels: contralateral presentation. (TIF) [file pone.0044910.s005.tif]

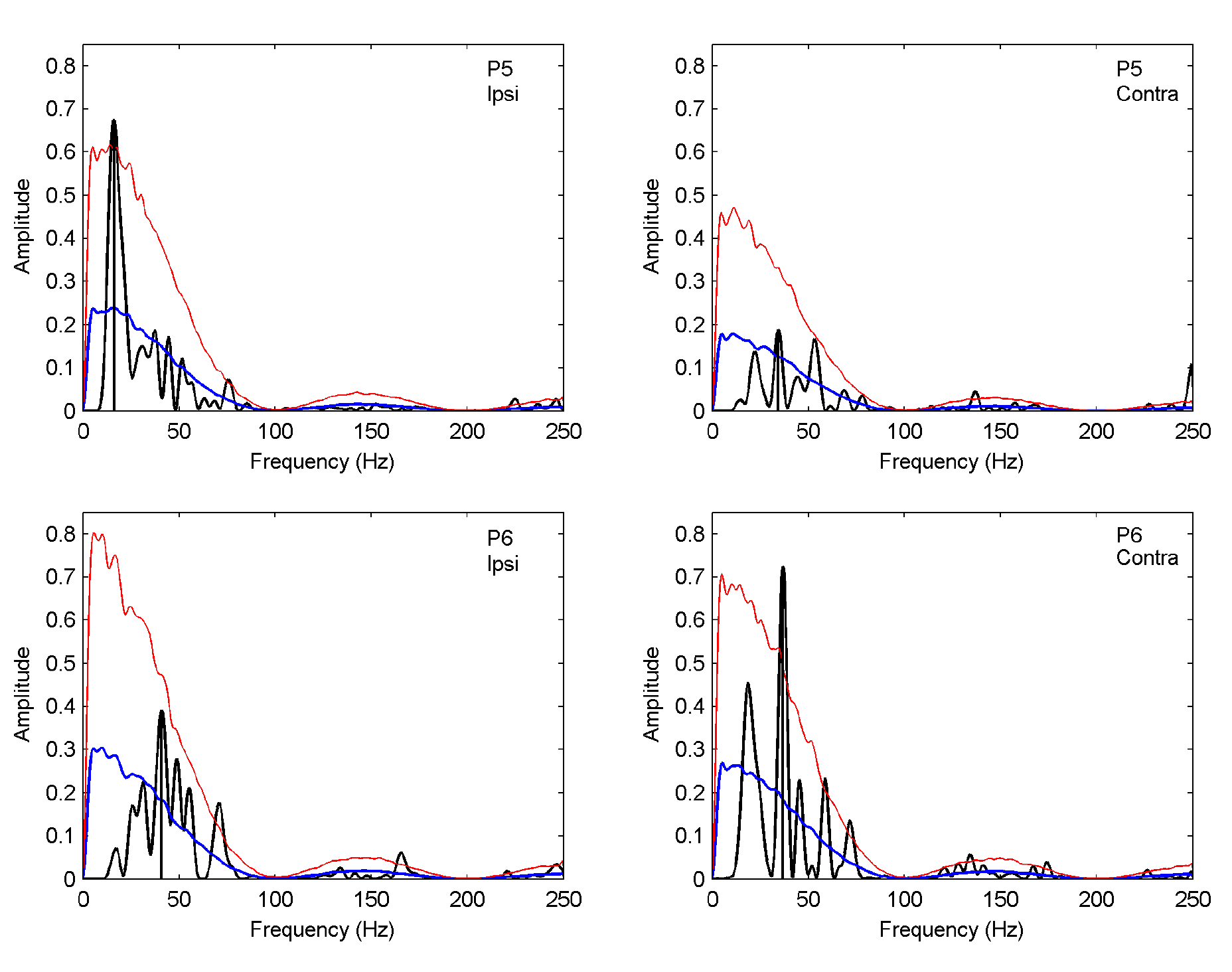

Supplement: Figure S6 — Spectrograms for participants P4, P5, and P6. Left panels: Ipsilateral presentation. Right panels: contralateral presentation. (TIF) [file pone.0044910.s006.tif]
