# Supplementary material for: Saccadic Reaction Times to Audiovisual Stimuli Show Effects of Oscillatory Phase Reset
Source: PLoS One. 2012 Oct 3;7(10):e44910. doi: 10.1371/journal.pone.0044910 (PMC3463580; doi:10.1371/journal.pone.0044910)
Supplement: Table S1 — Percentage of errors by type for each participant. (PDF) [file pone.0044910.s007.pdf]

Table 1. Percentage of errors by type for each participant.

	Participant					
Type of error	1	2	3	4	5	6
Saccades before any signal	0	0	0	0	0	0
Amplitude not within 3 std	0.9	2.1	1.6	1.1	1.9	2.0
SRT < 80	3.5	1.9	1.4	0.7	3.3	0.4
SRT > 500	0	0	0	0	0	0
Directional	0.9	1.1	0.5	0.5	1.3	0.1
Total	5.8	5.1	3.5	2.3	6.5	2.5
