# Supplementary material for: Saccadic Reaction Times to Audiovisual Stimuli Show Effects of Oscillatory Phase Reset
Source: PLoS One. 2012 Oct 3;7(10):e44910. doi: 10.1371/journal.pone.0044910 (PMC3463580; doi:10.1371/journal.pone.0044910)
Supplement: Text S1 — Probing for slower oscillatory activity. (PDF) [file pone.0044910.s008.pdf]

Probing for slower oscillatory activity

The trend component, $SRT_{Trend}(t)$, which was eliminated from the time series, $SRT(t)$, may itself include slow periodic components in modulating the SRT. To extract them, the trend of the trend function $SRT_{Trend}(t)$ was removed by a polynomial function of degree 2. Again, this was necessary because the long scaled structure of $SRT_{Trend}(t)$ was superimposing small ones and completely nullified the estimation of the frequency spectral content. Furthermore, the polynomial function of degree 2 was chosen since it roughly described the trend and does not represent a periodic structure.

Figure S3 shows the procedure, exemplified for participant P1 for ipsilateral stimulus presentation.

The power spectra and spectrograms for all participants are shown in Figures S4 and Figures S5 and S6, respectively. Maximal power could be observed between 7 Hz and 12 Hz, with a modus of 8 Hz. Similar to the higher frequency spectrograms, the lower frequency spectrograms showed some patterns of periodicity.
